# Resistin-induced stromal cell-derived factor-1 expression through Toll-like receptor 4 and activation of p38 MAPK/ NFκB signaling pathway in gastric cancer cells

**DOI:** 10.1186/1423-0127-21-59

**Published:** 2014-06-14

**Authors:** Yung-Yu Hsieh, Chien-Heng Shen, Wen-Shih Huang, Chih-Chien Chin, Yi-Hung Kuo, Meng Chiao Hsieh, Hong-Ren Yu, Te-Sheng Chang, Tseng-Hsi Lin, Yung-Wei Chiu, Cheng-Nan Chen, Hsing-Chun Kuo, Shui-Yi Tung

**Affiliations:** 1 Department of Hepato-Gastroenterology, Chang Gung Memorial Hospital, Chiayi, Taiwan; 2 Graduate Institute of Clinical Medical Sciences, College of Medicine, Chang Gung University, Taoyuan, Taiwan; 3 Division of Colon and Rectal Surgery, Department of Surgery, Chang Gung Memorial Hospital, Chiayi, Taiwan; 4 Department of Pediatrics, Chang Gung Memorial Hospital-Kaohsiung Medical Center, Graduate Insititute of Clinical Medical Science, Chang Gung University College of Medicine, Taoyuan, Taiwan; 5 Division of Hematology, Department of Internal Medicine, Taichung Veterans General Hospital, Taichung, Taiwan; 6 Department of Internal Medicine, School of Medicine, Chung Shan Medical University, Taichung, Taiwan; 7 Emergency Department and Hyperbaric Oxygen Therapy Center, Tungs’ Taichung MetroHarbor Hospital, Taichung, Taiwan; 8 Institute of Medicine, Chung Shan Medical University, Taichung, Taiwan; 9 Department of Biochemical Science and Technology, National Chiayi University, Chiayi, Taiwan; 10 Institute of Nursing and Department of Nursing, Chang Gung University of Science and Technology, Chiayi, Taiwan; 11 Chronic Diseases and Health Promotion Research Center, Chang Gung University of Science and Technology, Chiayi, Taiwan; 12 Research Center for Industry of Human Ecology, Chang Gung University of Science and Technology, Taoyuan, Taiwan; 13 Chang Gung University College of Medicine, Taoyuan, Taiwan

**Keywords:** Gastric cancer, Obesity, TLR4, NF-κB, Resistin

## Abstract

**Background:**

Stromal cell-derived factor-1 (SDF-1) (CXC chemokine ligand-12)/CXC chemokine receptor 4 (CXCR4) is involved in the carcinogenesis of human gastric cancer, where it stimulates angiogenesis and favors metastasis of tumor cells to distant organs. In addition, resistin is suggested to be an important link between obesity and the development of gastric cancer. Resistin has identified as an important player in inflammatory responses, and emerged as a mediator in inflammation-associated cancer. A limited number of studies have investigated the association of resistin and SDF-1 with gastric cancer. Herein, we investigated the molecular mechanisms by which resistin influences the expression of SDF-1 in gastric carcinoma cells.

**Results:**

Human gastric cancer cell lines were exposed to doses of resistin; SDF-1 expression and secretion levels were then determined. Real-time polymerase chain reaction and western blotting analyses were performed to clarify molecular changes. Inhibition of Toll-like receptor 4 (TLR4) by a competitive antagonist inhibited resistin-induced SDF-1 expression. Pharmacological inhibitors and small interfering RNA (siRNA) demonstrated that activation of the p38 mitogen-activated protein kinase (MAPK) pathway is critical for resistin-induced SDF-1 expression mediated by TLR4. The promoter activity and transcription factor enzyme-linked immunosorbent assay revealed that resistin induced expression of SDF-1 mediated by NF-κB in gastric cancer cells. Inhibition of p38 MARK activation blocked the SDF-1-induced expression and the SDF-1 promoter activity in the cancer gastric cells. Chromatin immunoprecipitation assay revealed that inhibition of p38 MARK activation also blocked the resistin-increased NF-κB-DNA-binding activity.

**Conclusions:**

Resistin-induced SDF-1 upregulation by activation of TLR4, p38 MARK and NF-κB may explain a new role of resistin in the link of obesity and gastric cancer.

## Background

Gastric cancer ranks as the fourth most frequent of malignant tumors [1]. Although it is the second or third most frequent cause of cancer death worldwide [2], its etiology is not well understood. The disease is often the result of long-term interactions of many factors relating to individual habits, diet, environment, and genetics, as well as chronic gastritis, gastric polyps, gastric dysplasia, post-surgery gastric remnants, and long-term infection by *Helicobacter pylori*[3]. *H. pylori* is recognized as a causative factor of chronic gastritis, gastroduodenal ulcers, gastric cancer, and mucosa-associated lymphatic tissue lymphoma. In previous study, chronic gastritis related human diseases, including gastroduodenal ulcers, gastric cancer, and mucosa-associated lymphatic tissue lymphoma, were found to result from an excessive formation of epithelial cells or gastric mucin injury and inflammation caused by chronic *H. pylori* infection [4]. Several studies have indicated that the typical LPSs recognized by the Toll-like receptor 4 (TLR4) complex expressed on host cells, contributing to activation of the p38 mitogen-activated protein kinase (MARK) pathway [5,6].

Obesity is a risk factor for gastric cancer mainly because obesity enhances the incidence of gastroesophageal reflux, which may damage the mucosa around the gastric cardia, resulting in an increased likelihood of cancer. Obesity is defined as a very high ratio of body fat to other tissue [7]. Too much body fat has a significant impact on health, particularly in terms of insulin resistance. The proteins secreted by the fat tissues play a very important role in regulating metabolism [8]. The regulation of resistin in carbohydrate metabolism is considered to be associated with insulin resistance. Resistin is capable of regulating the insulin-stimulated metabolism of carbohydrates in many insulin target organs [9]. Since the discovery of resistin, most studies have focused on the relationship of resistin to obesity and diabetes. An increasing number of studies have focused on the role of resistin in cancer development, and the impact of resistin on inflammation is the focus of several current academic studies [10,11]. These studies have found that resistin is negatively correlated with high density lipoproteins in diabetic patients; however, resistin is positively correlated with C-reactive protein (CRP), an indicator of inflammation [12]. This association indicates that higher levels of the resistin may be related to inflammatory responses. In addition, studies have found that adipose tissue is not the exclusive source of resistin; large amounts of resistin and resistin-like molecules are also found in non-adipose tissues under inflammation [13]. Inflammatory response can release interleukin (IL)-6, IL-8, IL-1β, and tumor necrosis factor alpha (TNFα) through the NF-κB pathway [11]. Thus far, no study has investigated the association of resistin and any known receptor to activate downstream MAPK kinase that further activate nuclear factor-κB (NF-κB p50/p65) in human gastric cancer.

Chemoattractant proteins are a group of small proteins of molecular weight ranging from 8 to 12 kDa that can be induced by inflammatory substances to release into the extracellular environment. More than 40 types of human cell chemoattractant proteins have been identified [14]. Chemoattractant proteins have a number of functions such as inducing the movement, growth, and differentiation of white blood cells. These inflammatory responses are closely related to gastric cancer [15]. One of the causative factors of inflammatory responses is the production and induction of chemoattractant proteins. Previous studies have found that the stromal cell-derived factor-1 (SDF-1) can regulate cancerous cell movement and blood vessel regeneration via its specific receptors CXCR4 and CXCR7 [16]. Gastric inflammation is an integral step in gastric cancer development; therefore, factors inducing and regulating responses to inflammation may play a key role in gastric cancer prognoses [17]. From this viewpoint, because chemokines have certain roles in microbial immune and inflammation responses, the resistin-induced secretion of SDF-1 may be correlated to the control of gastric cancer.

Gastric cancer can be correlated with obesity. Researchers have pointed out that resistin is the blood biological indicator of gastric cancer and is related to patient prognosis [18]. Moreover, SDF-1 acts in cancerous cells as a growth and survival factor; however, the implication of resistin stimulation by the chemoattractant SDF-1 has not been studied. In the present study, we investigated whether resistin stimulates the expression of SDF-1 by activating the p38 MAPK intracellular signaling cascades and the transcription factors NF-κB and p50. Our findings provide evidence of the molecular mechanisms of SDF-1 expression and its secretion by resistin via a TLR4-dependent pathway in gastric cancer cells.

## Methods

### Chemical reagents and antibodies

All culture materials were purchased from Gibco (Grand Island, NY). 3-(4,5-dimethylthiazol-2-yl)-2,5-diphenyltetrazolium bromide (MTT), PD98059 (MEK1 inhibitor), SP600125 (JNK inhibitor), SB203580 (p38 inhibitor), SN50, and PDTC (pyrrolidinedithiocarbamate ammonium) were purchased from Sigma (St. Louis, MO). Mouse monoclonal antibodies against p38 MARK (9 F12) and phospho-p38 MARK (D-8) were purchased from Santa Cruz Biotechnology (Santa Cruz, CA). Human CXCL12/SDF-1 enzyme-linked immunosorbent assay (ELISA) kit was obtained from Cell Sciences (Canton, MA). ERK siRNA (ordering number: s11137 and s11143), JNK siRNA (ordering number: 1452 and s11152), p38 siRNA (ordering number: 1312), p50 siRNA (ordering number: 5121), p65 siRNA (ordering number: s11916), and control siRNA (scrambled negative control containing random DNA sequences) were purchased from Invitrogen (Carlsbad, CA). TLR4 siRNA was purchased from Sigma-Proligo (Singapore). The bacterial lipopolysaccharide from *Rhodobacter sphaeroides* (LPS-RS, TLR4 antagonist) was obtained from Invivogen (San Diego, CA).

### Cell culture

The gastric carcinoma cell line TSGH 9201 and AGS cells was purchased from the Bioresources Collection and Research Center (BCRC) of the Food Industry Research and Development Institute (Hsinchu, Taiwan). Cells were maintained in RPMI 1640 supplemented with 10% fetal bovine serum (FBS) and 1% penicillin/streptomycin in a CO_2_ incubator at 37°C.

### Real-time quantitative PCR

Real-time PCR was performed using an ABI Prism 7900HT with the FastStart DNA SYBR Green I kit (Roche Diagnostics GMbH, Mannheim, Germany). The designed primers in this study were SDF-1 forward primer, 5'- ATTCTCAACACTCCAAACTGTGC-3'; SDF-1 reverse primer, 5'- ACTTTAGCTTCGGGTCAATGC-3'; 18S rRNA forward primer, 5'-CGGCG ACGAC CCATT CGAAC-3'; and 18S rRNA reverse primer, 5'-GAATC GAACC CTGAT TCCCC GTC-3'. Quantification was performed using the 2^−ΔΔCt^ method [19]. All samples were measured in duplicate. The average value of the duplicates was used as the quantitative value.

### ELISA

CXCL12/SDF-1 expression on the cancer cell surface was measured by ELISA as previously described [19]. Release of SDF-1 into culture media was analyzed using commercially available ELISA kit purchased from Cell Sciences (Canton, MA). The assays and data calculations were performed according to the manufacturer’s instructions.

### Preparation of total cell extracts and immunoblot analyses

TSGH 9201 cells were lysed with a buffer containing 1% NP-40, 0.5% sodium deoxycholate, 0.1% sodium dodecyl sulfate (SDS), and a protease inhibitor mixture (phenylmethylsulfonyl fluoride, aprotinin, and sodium orthovanadate). The total cell lysate (50 μg of protein) was separated by SDS-polyacrylamide gel electrophoresis (PAGE) (12% running, 4% stacking) and analyzed by using the designated antibodies and the Western-Light chemiluminescent detection system (Bio-Rad, Hercules, CA), as previously described [20].

### DNA plasmid, siRNA, transfection, and luciferase assay

Human SDF-1 promoter constructs containing −1010/+30, −630/+30, −430/+122, −214/+30, −121/+30, and −20/+30 of SDF-1 5'-flanking DNA linked to the firefly luciferase reporter gene of plasmid pGL4 (Promega, Madison, WI) were used as previously reported [21]. DNA plasmids at a concentration of 1 mg/ml were transfected into TSGH 9201 cells by Lipofectamine (Gibco, Carlsbad, CA). The pSV-β-galactosidase plasmid was cotransfected to normalize the transfection efficiency. For siRNA transfection, TSGH 9201 cells were transfected with the designated siRNA using an RNAiMAX transfection kit (Invitrogen, Carlsbad, CA) [19]. The effectiveness of the silencing was validated: ERK-, JNK-, p38 MARK-, p65-, and p50-specific siRNAs (compared with control siRNA) caused at least 80% reduction in the protein expression of ERK, JNK, p38 MARK, p65, and p50, respectively. The cells were transfected with the specific TLR4 siRNA (CGAUGAUAUUAUUGACUUA[dT]; [dT]UAAGUCAAUAAUAUCAUGG[dT][dT]).

### NFκB p50 transcription factor assay (TF ELISA assay)

Nuclear extracts of cells were prepared by nuclear protein extract kit (Panomics, Redwood City, CA). Equal amounts of nuclear proteins were used for quantitative measurements of NF-κB p50 activation using commercially available ELISA kit (Panomics, Redwood City, CA) that measure p50 DNA-binding activities [19].

### Chromatin immunoprecipitation assay (ChIP)

The ChIP assay was carried out as previously described and ChIP assay kit used was from Upstate Biotechnology (Lake Placid, NY) [19]. Cells were fixed with 1% formaldehyde, washed, then harvested in SDS lysis buffer. After sonication, lysates containing soluble chromatin were immunoprecipitated using 2 μg of antibody against p50. DNA was purified with a PCR Purification Kit (QIAGEN, Venlo, The Netherlands). The resulting DNA was used for PCR analysis, and the amplified DNA fragments were visualized on an agarose gel. PCR was performed with the following primers that amplify the parts of the human SDF-1 promoter that contain the p50 binding sites from −669 ~ −569: 5'- GTTTCCACAGGCGAATGG -3' and 5'- GGACCTCACAGCCTCAAGTC -3'.

### Statistical analysis

The experiments were performed in triplicate independent experiments, and data were presented as three repeats from one independent experiment. Data were reported as the mean ± standard deviation or standard error of the mean and evaluated by one-way analysis of variance. SPSS version 16.0 (SPSS, Inc., Chicago, IL) was used for all statistical analyses. Significant differences were established at *P* < 0.05.

## Results

### Effect of resistin on expression of SDF-1 in gastric carcinoma TSGH 9201 and AGS cells

**Figure 1:**
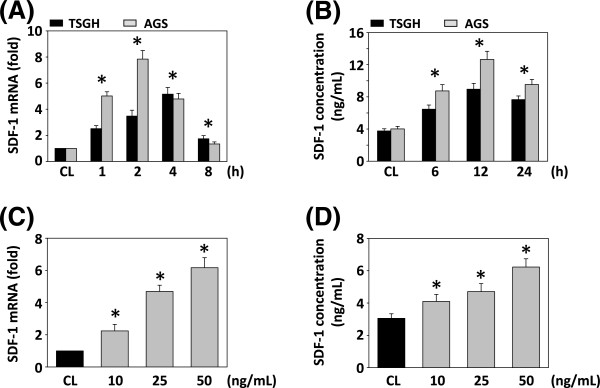
**Induction of SDF-1 expression in human gastric carcinoma cell line TSGH 9201 and AGS cells by resistin stimulation.** TSGH 9201 and AGS cells were treated with vehicle and resistin treatment. RNA samples were isolated at the indicated time periods or doses. All bar graphs represent folds of control cells (CL) and normalized to 18S rRNA by real-time PCR analysis **(A, C)**. Human SDF-1 protein secretion was determined by ELISA analyses **(B, D)**. Cells were stimulated with 25 ng/mL resistin for the times indicated **(A, B)**, or induced at various doses for 4 h **(C)** or 6 h **(D)**. RNA levels were quantified with the control being set at 100%. The experiments were performed in triplicate, and data are presented as means ± SD. The symbol *indicates means that are significantly different when compared to the control group (CL) of TSGH 9201 with *P* < 0.05, respectively.

To determine whether SDF-1 is induced by resistin, we exposed the human gastric cancer cell lines TSGH 9201 and AGS to a range of resistin doses and performed experimental assays. Cells were exposed to a 25 ng/mL dose of resistin for the indicated times. The changes in SDF-1 mRNA expression were analyzed by real-time PCR; SDF-1 secretion in conditioned media was detected by ELISA. The SDF-1 mRNA reached its highest level at 4 h of resistin stimulation (Figure 1A). The secretion of SDF-1 protein began to increase after resistin treatment and reached its highest level at 6 h (Figure 1B). In addition, the resistin-induced SDF-1 mRNA expression and protein secretion in TSGH 9201 cells was dose dependent (Figure 1C-D). The results demonstrate that resistin significantly induced gene expression. Based on our results, it is possible that in gastric carcinoma cell, resistin induced pathway-related proteins may be studied as potential markers in terms of the prediction of response to treatment or prognosis. Further investigation, we used TSGH 9201 Cell to evaluate the effect of resistin on other pro-tumoral CXC chemokines gene expression. Our data demonstrate that resistin significantly induced-related gene expression, such as GRO, ENA78, GCP-2 or IL-8 (Additional file 1: Figure S1).

### Resistin-induced SDF-1 expression in gastric cancer is mediated by p38 MAPK

**Figure 2:**
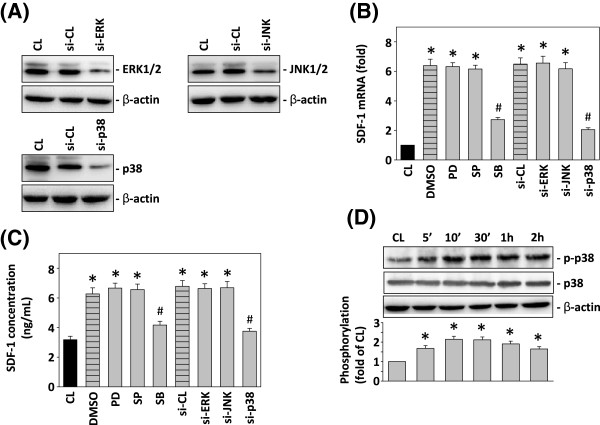
**p38 MAPK pathway is required for resistin-stimulated SDF-1 expression. (A)** TSGH 9201 cells were incubated with various concentrations of the specific MEK1 inhibitor PD98059 (PD, 10 μM), the c-Jun N-Terminal Kinase (JNK/SAP Kinase) Inhibitors SP600125 (SP, 10 μM), or the p38 Inhibitor SB 203580 (SB, 10 μM) for 1 h, transfected with control siRNA (si-CL), control pcDNA3 vector (vec), or a specific siRNA of si-ERK, si-JNK, or si-p38. Next, the cells were treated with 25 ng/mL resistin for 4 h **(B)** or 6 h **(C)**. **(B)** All bar graphs represent folds of CL group and normalized to 18S rRNA. **(C)** Human SDF-1 secretion was determined by ELISA. The results are shown as mean ± SEM. *P < 0.05 versus CL. #*P* < 0.05 versus vehicle control (DMSO) or control siRNA (si-CL) with resistin stimulation. **(D)** Total cell lysates of cells treated with or without resistin for the indicated time were extracted, and the phosphorylated proteins of p38 MAPK, and p38 MARK were immunodetected as described in “Materials and Methods”. Protein levels were quantified by densitometric analysis, with the control being set at 100%.

To clarify the events of resistin-induced SDF-1 expression, we analyzed specific MAPK siRNAs to determine the signaling pathways associated with resistin-induced SDF-1 expression in TSGH 9201 cells (Figure 1A). As shown in Figure 2B and C, the mRNA level and secretion of SDF-1 were increased by the resistin stimulation, and they were significantly inhibited by SB203580, but not by PD98059 or SP600125. To further confirm the involvement of p38 MAPK, but not ERK and JNK, in the modulation of the SDF-1 expression by resistin induction, we examined the effects of specific MAPK siRNAs of these signaling pathways on resistin-induced SDF-1 expression in TSGH 9201 cells (Figure 2A). The resistin-induced SDF-1 mRNA expression (Figure 2B) and SDF-1 secretion (Figure 2C) were inhibited by transfection with p38- siRNA, but not by transfection with ERK-, JNK-, and control siRNAs (100 mmol/ml for each). These data suggest that the p38 MAPK pathway is involved in regulating the resistin-induced SDF-1 expression in gastric cancer cells. To determine the effect of resistin on the activation of the kinase signaling pathway, we assessed whole cell lysates from resistin-treated TSGH 9201 cells by Western blotting analysis using antibodies against activated Phospho-p38 MAPK and p38 MAPK. As shown in Figure 2D, the treatment of TSGH 9201 cells with resistin resulted in the time-dependent phosphorylation of p38 MAPK within 2 h. SDF-1 expression analysis revealed that the resistin induction is mediated by the p38 MAPK-dependent pathway in TSGH 9201 cells.

### TLR4 regulates resistin-induced expression of SDF-1 and promoter activity

**Figure 3:**
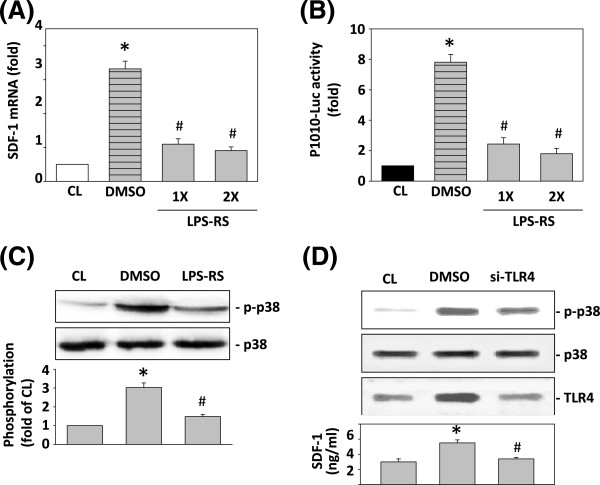
**Effect of the TLR4 antagonist in blocking resistin-induced expression of SDF-1 and p38 MAPK. (A)** TSGH 9201 cells were kept as control (CL) or stimulated with 25 ng/mL resistin. Cells were pretreated with or without 1 or 2 mg/ml LPS-RS for 1 h and then stimulated with 25 ng/mL resistin for 4 h. SDF-1 mRNA levels were determined through real-time PCR in TSGH cells and normalized to 18S rRNA. **(B)** SDF-1-1010-Luc activity were determined in TSGH cells with or without 1 or 2 mg/ml LPS-RS for 1 h. **(C)** TSGH cells were kept as CL or stimulated with resistin for 30 min, and the phosphorylation of p38 was determined by western blotting. All bar graphs represent folds of CL cells. *P < 0.05 versus CL. #*P* < 0.05 versus vehicle control (DMSO). **(D)** TSGH 9201 cells were transfected with control siRNA (si-CL) or a specific siRNA of si-TLR4 and the cells were treated with 25 ng/mL resistin for 30 min and 6 h, and then the phosphorylation of p38 and TLR4 were determined by western blotting. Human SDF-1 secretion was determined by ELISA.

To assess the role of TLR4 in the resistin-induced SDF-1 expression in TSGH 9201 cells, we demonstrated the effect of the TLR4 antagonist (LPS-RS, complete competitive inhibition) on the resistin-induced SDF-1 expression and the promoter activity. Pretreatment with LPS-RS significantly inhibited the expression of SDF-1 mRNA in TSGH 9201 cells (Figure 3A). To evaluate whether inhibition of the SDF-1 expression by the MAPK signaling pathway occurs at the transcriptional level, we compared unstimulated cells to those treated with resistin. The treatment with resistin increased the luciferase activity 8.0 fold compared with the unstimulated cells after normalization through transfection control. Pretreatment of cells with LPS-RS for 2 h resulted in a marked 1.8- to 2.2-fold inhibition of the resistin-induced SDF-1 p1010-Luc promoter activity (Figure 3B). To evaluate whether the SDF-1 expression by TLR4 involved the MAPK signaling pathway at the transcriptional level, we compared control cells to those stimulated with resistin for 30 min. LPS-RS significantly inhibited the resistin-induced phosphorylation of p38 MAPK after 2 h (Figure 3C). Furthermore, TSGH 9201 cells were transfected with the TLR4 siRNA, and the phosphorylation of p38 MAPK and the SDF-1 expression were then examined. Figure 3D indicates the effectiveness of TLR4 siRNA on p38 MAPK and SDF-1expression after resistin stimulation.

### NF-κB is necessary for resistin induction of human SDF-1 promoter activity

**Figure 4:**
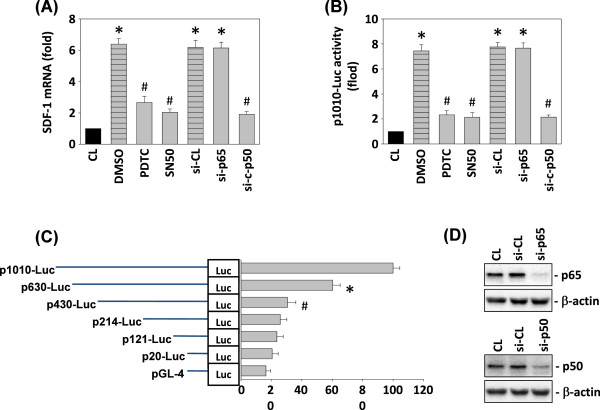
**Effect of NFκB in resistin-induced SDF-1 mRNA expression and promoter activity. (A)** SDF-1 mRNA and **(B)** SDF-1-1010-Luc activity were determined in TSGH 9201 cells pretreated with vehicle (DMSO), NF-κB inhibitor Pyrrolidinedithiocarbamate Ammonium (PDTC), or peptides (SN50), transfected with control siRNA (si-CL), si-p65, or si-p50), and then stimulated with 25 ng/mL resistin for 4 h. SDF-1 mRNA levels were determined through real-time PCR in TSGH cells and normalized to 18S rRNA. **(C)** SDF-1-1010-Luc activity were determined in TSGH cells with or without resistin for 2 h. The SDF-1 promoter p1010-Luc plasmid and several deletion promoter constructs. Right panel: TSGH9201 cells were cotransfected with 5′-deletion constructs and stimulated with 25 ng/mL resistin for 2 h. SDF-1 promoter activity was measured using a luciferase assay normalized to β-galactosidase activity and was shown to be relative to that of TSGH cells transfected with p1010-Luc (set to 100%). All bar graphs represent folds of CL cells. *P < 0.05 versus CL (Vehicle control, DMSO and p1010-Luc). **(D)** TSGH 9201 cells were transfected with control siRNA (si-CL), or a specific siRNA of si-p65 and si-p50 and then the cells were treated with 25 ng/mL resistin for 6 h. Total cell lysates were extracted, and the proteins of p65, p50 and β-actin were immunodetected.

**Figure 5:**
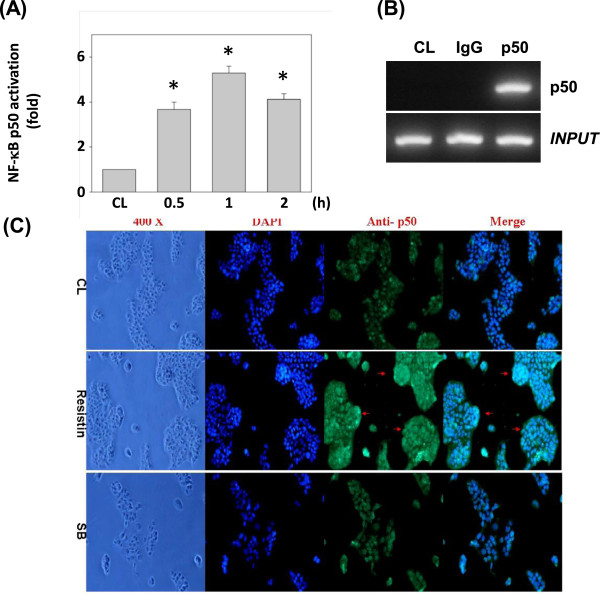
**NFκB binding activities of the SDF-1 promoter region induced by resistin induction. (A)** NFκB p50 activation was determined by a TF ELISA assay for 2 h in TSGH 9201 cells . All bar graphs represent folds of control cells (CL), mean ± SEM. *P < 0.05 versus CL. **(B)** ChIP assay was performed for p50 by using p50 antibody. **(C)** NFκB p50 translocations in nuclei by immunofluorescent staining. TSGH-9201 cells treated with 50 nM resistin or 0.2% DMSO (as control) for 12 h were fixed, permeabilized, and stained with anti-p50, and then observed by phase contrast microscopy (left panels) and fluorescence microscopy. Arrows indicate change and nuclear location.

The human SDF-1 gene promoter contains multiple transcription binding sites. To determine the *cis*-acting elements in the SDF-1 gene promoter that mediate resistin-induced SDF-1 transcription, a luciferase assay was applied using the p1010-Luc plasmid and several deletion promoter constructs (Figure 4A). To clarify the binding region of the SDF-1 promoter, we constructed and analyzed a series of 5′-deletion mutants. In TSGH 9201 cells, the −1010/+30 region of SDF-1 directed maximum luciferase activity. The sequence deletion from −1010 to −430 (NF-κB binding sites) caused luciferase activity to decline to about 30%, nearly abolishing the activity (Figure 4A).Further, we assayed whether NF-κB activation was involved in resistin-induced SDF-1 gene expression. TSGH 9201 cells were transfected with p65 or p50 siRNA, or incubated with specific inhibitors of NF-κB (SN50, 50 μM or PDTC, 50 μM) for 1 h, followed by stimulation with resistin for 4 h. The resistin-induced SDF-1 mRNA expression (Figure 4B) and SDF-1 p1010-Luc promoter activity (Figure 4C) were significantly inhibited by SN50, PDTC, or siRNA p50, indicating that NF-κB p50 is involved in regulating SDF-1 gene induction.To investigate whether p50 binds the SDF-1 promoter region in TSGH 9201 cells, we performed quantitative analysis to determine the binding activity of NF-κB p50 using TF ELISA kits (Figure 5A). The results showed that treating TSGH 9201 cells with resistin raised the binding activity of p50 DNA within 2 h. To confirm these results, ChIP analysis was performed in vitro. Immunoprecipitated chromosomal DNA with anti-p50 antibody was subjected to PCR using primers designed to amplify the SDF-1 promoter region (−1010 to −430) harboring the p50 binding sites. NF-κB p50, but not the control antibody, did indeed bind to the SDF-1 promoter region (Figure 5B). These data suggest that these sequences were indeed p50 binding sites. We used double labeling of p50 and DAPI to evaluate the effect of resistin in TSGH 9201 cells at 12 h. Representative immunoreactivity for phase contrast microscopy, DAPI (blue), p50 (green), and overlays in the TSGH cells (Figure 5C).

### MAPK signaling pathways are involved in resistin-induced SDF-1 promoter activity

**Figure 6:**
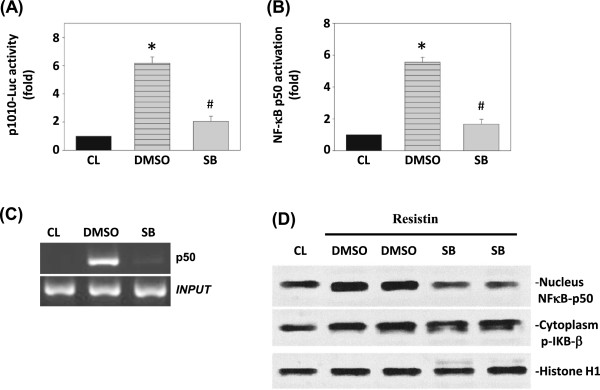
**p38 MAPK and NFκB signaling pathways are involved in resistin-induced SDF-1 promoter activity. (A)** SDF-1-1010-Luc activity was demonstrated in TSGH 9201 cells pretreated with vehicle (DMSO) and SB203580 (SB) individually 1 h, and then stimulated with 25 ng/mL resistin for 1 h. **(B)** NFκB p50 activation was determined by TF ELISA assays in TSGH 9201 cells pretreated with vehicle (DMSO) and SB203580 (SB) individually or in combination for 1 h, and then treated with resistin for 1 h. All bar graphs represent folds of CL in TSGH 9201 cells, mean ± SEM. *P < 0.05 versus CL. #*P* < 0.05 vs. vehicle control (DMSO). **(C)** NFκB p50 binding to SDF-1 promoter in TSGH 9201 cells after 1 h resistin stimulation was determined by ChIP assay. **(D)** TSGH 9201 cells were incubated with the p38 inhibitor SB 203580 (SB, 10 μM) for 1 h. Next, the cells were treated with 25 ng/mL resistin for 12 h. Effect of resistin on the phosphorylation of IκBβ as well as on p50 nuclear translocation. Nuclear and cytoplasm cell lysate proteins were prepared and analyzed by western blot.

Members of the MAPK family have been implicated in the regulation of gene expression by resistin [22]. To evaluate the induction of SDF-1 expression by MAPK signaling pathways through the transcriptional level, TSGH 9201 cells were incubated with a specific inhibitor of p38 MAPK (SB203580) for 1 h before and during stimulation with resistin, and the SDF-1 promoter activity and ChIP were analyzed. The data clearly demonstrated that pretreatment of cells with SB203580 resulted in marked inhibition of the resistin-induced SDF-1 promoter activity (Figure 6A). In addition, SB203580 significantly inhibited both resistin-induced p50 activation (Figure 6B) and NF-κB p50-DNA binding activity (Figure 6C). We have used TSGH 9201 cells to evaluate the effect of resistin on phosphorylation of IκBβ as well as on p50 nuclear translocation. Our data demonstrate that resistin significantly induced p50 expression in TSGH 9201 cells via p38 MAPK. Taken together, these results showed that p38 MARK signaling pathway are involved in the resistin-induced SDF-1 expression (Figure 6D).

## Discussion

Obesity has been associated with lower rates of survival in patients with gastric cancer [23]. Adipocytokines such as TNFα, IL-6, adiponectin, leptin, visfatin, and resistin are cytokines secreted primarily by visceral adipose tissue and are thought to be involved in the positive correlation between obesity and the increased risk of gastric cancer [24]. On the other hand, several observers have suggested that resistin mediates the induction of inflammation in both adipose and non-adipose tissue [25]. The elevation of resistin and its role in inflammation in the intestine has resulted in the release of cytokines through the TLR4/ NF-κB pathway [26-28]. Recent studies have demonstrated the essential role of the resistin cascade, and a higher expression of resistin was evident in intestinal-type gastric carcinomas with tumor differentiation, tumor invasion, and lymph node metastasis [13]. The essential role of resistin, as well as its association with gastric cancer, make it a factor of concern as well as a potential a biomarker for gastric cancer progression [18]; therefore, it is clinically relevant to study the mechanism by which resistin influences tumor cells. In this study, we evaluated the molecular mechanisms underlying the roles of resistin in controlling SDF-1 expression in gastric cancer cells. SDF-1 was upregulated by resistin stimulation in TSGH 9201 cells. Resistin-induced expression of SDF-1 was mediated by the p38 MAPK and NF-κB pathways, and interaction between resistin and TLR4 was required for resistin-induced intracellular signaling and SDF-1 expression.

SDF-1 also promotes tumor development by stimulating angiogenesis and by processing the metastasis of CXCR4-positive tumor cells to distant organs producing SDF-1 [27]. Studies have shown that the level of plasma SDF-1 was higher in the high- incidence cancer group. In addition, SDF-1 modulates the angiogenic process directly (by binding to receptors CXCR4 expressed on endothelial cells) or indirectly (by inducing the secretion of matrix-metalloproteases or angiogenic factors) [29]. It has been suggested that SDF-1 is produced by gastric tumor cells themselves and can act on the tumor cells in a paracrine or autocrine fashion [30]. In summary, our study demonstrated that the effect of resistin on TSGH 9201 cells was mediated by the activation of p38 MAPK and that NF-κB transcriptional activation plays an important role in the expression of SDF-1. TF ELISA and luciferase assays demonstrated a resistin-induced increase in the NF-κB- and DNA-binding activity. In contrast, the inhibition of NF-κB and p38 MAPK activation in TSGH 9201 cells after treatment with NF-κB and p38 MAPK inhibitors and transfection with specific p50 and p38 siRNAs prohibited the resistin-induced expression and secretion of SDF-1.

The promoter region of the SDF-1 gene has several transcriptional factor binding sites [21]. This study demonstrated the mechanism by which resistin induces SDF-1 gene expression of gastric cancer cells. The important findings are as follows: the expression of SDF-1 is mediated by the NF-κB p50 pathway. Construction and analyses of 5′-deletions in the −1010 to −430 region of the SDF-1 promoter showed that the activity decreased to 30% and was nearly abolished. ChIP DNA with anti-p50 antibody that was subjected to PCR analysis showed the SDF-1 promoter region (−1010 to −430) harboring the NF-κB p50 binding sites. NF-κB proteins are members of a superfamily of transcription factors whose activities play a crucial role in cellular activation, proliferation, and apoptosis, which can be triggered through the MAPK pathway in gastric cancer cells [31]. During the early stages of invasion and metastasis of carcinoma cells, p38 MAPK plays a key role [5,32,33]. In our present study, we found that the gastric cell line, TSGH 9201, persisted in expressing activated p38 MAPK after exposure to resistin and high levels of this kinase are associated with an increased capacity to induce the binding of NF-κB p50 to the promoter region of SDF-1.

Previous data suggest that regulation of TLR receptors in gastric carcinogenesis might go beyond *H. pylori* infection, and is thought to be associated with tumor cancers [34]. Resistin has been reported to be significantly correlated with stage progression of gastric cancer [18,35]. We investigated the role of resistin signaling factors downstream of the p38 MARK and NF-κB activation sites that lead to SDF-1 transcriptional activation in TSGH 9201, and the pathophysiological implication of the role of resistin in gastric cancer should be further explored.

## Conclusion

Taken together, our data suggest the mechanism by which resistin induces SDF-1 expression in gastric cancer cells. We found that treatment of gastric cancer cells with resistin resulted in the activation of signaling pathways mediated by TLR4. Further studies are required to explore the potential role of the resistin/ TLR4 axis as an effective therapeutic agent against gastric cancer.

## Competing interests

The authors declare that they have no competing interests.

## Authors’ contributions

Y-Y H: Provision of study material, collection and assembly of data, C-H S: Design, collection, assembly of data and manuscript writing, Wen-Shih Huang: Conception, collection, and assembly of data, C-C C: Provision of study material or patients, Y-H K: Provision of study material or patients, M C H: Provision of study material, collection, and assembly of data, H-R Y: Administrative support, collection, and assembly of data (flow cytometry), T-S C: Provision of study material or patients, T-H L: Administrative support, provision of study material or patients, Y-W C: Methodology design, administrative support, C-N C: Conception and design, collection and assembly of data, H-C K and S-Y T: Conception and design, financial support, administrative support, manuscript writing, final approval of manuscript. All authors read and approved the final manuscript.

## Supplementary Material

Additional file 1: Figure S1 Effect of resistin 50 ng/mL on expression of pro-tumoral CXC chemokines in gastric carcinoma TSGH 9201 at 4 h. Click here for file
