# Supplementary material for: Resistin-induced stromal cell-derived factor-1 expression through Toll-like receptor 4 and activation of p38 MAPK/ NFκB signaling pathway in gastric cancer cells
Source: J Biomed Sci. 2014 Jun 14;21(1):59. doi: 10.1186/1423-0127-21-59 (PMC4089564; doi:10.1186/1423-0127-21-59)

Effect of resistin 50ng/mL on expression of pro-tumoral CXC chemokines in gastric carcinoma TSGH 9201 at 4h

IL-8 mRNA expression

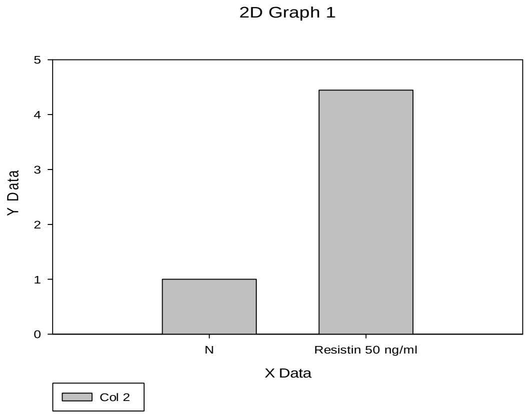

GCP-2 mRNA expression

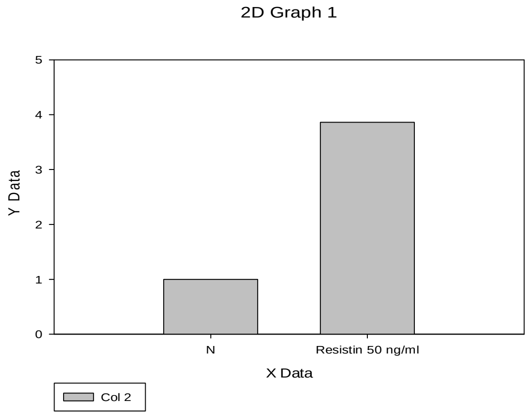

GRO mRNA expression

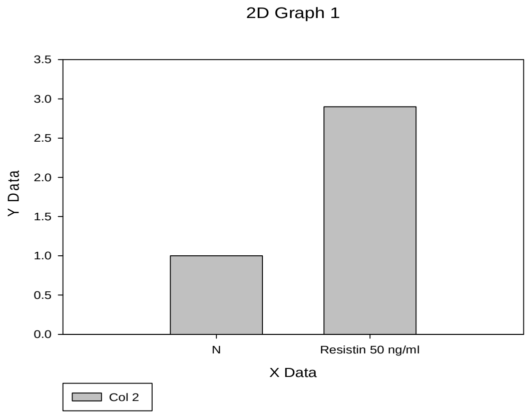

ENA-78 mRNA expression

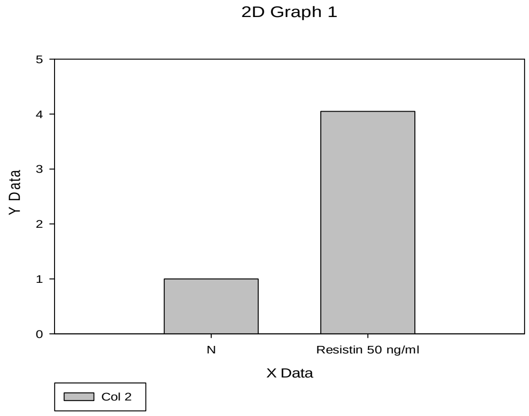

Supplement: Supplementary file 1 [file 1423-0127-21-59-S1.doc]
